# Cold-Tolerant Bacteria Isolated from Alpine Plants Can Promote Growth and Mitigate Cold Stress in Tomato Seedlings by Complex Transcriptional Reprogramming of Stress-Related Genes

**DOI:** 10.3390/plants14213316

**Published:** 2025-10-30

**Authors:** Irma Milanese, Aureliano Bombarely, Malek Marian, Michele Perazzolli

**Affiliations:** 1 Center Agriculture Food Environment (C3A), University of Trento, Via E. Mach 1, San Michele all’Adige, 38098 Trento, Italy; 2 Research and Innovation Centre, Fondazione Edmund Mach, Via E. Mach 1, San Michele all’Adige, 38098 Trento, Italy; 3 Department of Biotechnology and Plant Breeding, Institute of Plant Molecular and Cellular Biology (IBMCP, CSIC-UPV), Ciudad Politécnica de la Innovación, Ingeniero, Av. Fausto Elio s/n, 46022 Valencia, Spain

**Keywords:** cold stress, cold-tolerant bacterial endophytes, transcriptomics, RNA-Seq, oxidative stress, plant growth-promotion, cold tolerance

## Abstract

Cold stress adversely affects crop growth, and climate change is increasing its severity and frequency in many agricultural regions. Tomato plants are sensitive to low temperatures, although they activate some stress response mechanisms. Beneficial microorganisms can enhance cold-stress acclimation in tomato plants, but the transcriptional regulation underlying this process remains poorly understood. This study aimed to investigate the transcriptional processes activated by cold stress in tomato plants following inoculation with cold-tolerant bacteria isolated from alpine plants to identify genes potentially involved in cold stress acclimation. Among 41 cold-tolerant bacterial isolates tested, *Chryseobacterium* sp. GRCS301 and *Pseudomonas* sp. GRCS202 inoculation in sterilized soil promoted tomato growth under controlled non-stress (25 ± 2 °C) and cold-stress (10 ± 2 °C) conditions. Bacterial inoculations lowered H_2_O_2_ content and affected the transcriptional regulations activated in tomato shoots after one day and 14 days of incubation under cold-stress conditions. In mock-inoculated plants, cold stress downregulated genes related to energy generation, photosynthesis, and reproductive processes, highlighting its detrimental effects. Conversely, plants inoculated with *Chryseobacterium* and *Pseudomonas* upregulated genes involved in DNA replication, galactose metabolism, polysaccharide metabolism, photosynthesis, and protein metabolism in response to cold stress. Bacterial inoculation induced the expression of genes involved in reactive oxygen species homeostasis, cold-stress response, and hormonal signaling, suggesting that cold-tolerant bacteria trigger key transcriptional changes in tomato plants and enhance cold-stress acclimation.

## 1. Introduction

Cold stress represents one of the most common environmental factors that can negatively affect plant growth, plant development, and agricultural productivity worldwide [1]. Climate change intensifies the impact and frequency of cold stress on plants, since it is responsible for increased cold-air outbreaks and cold duration [2]. Moreover, global warming is responsible for mild winters and warm springs that can trigger premature phenological events [3]. For example, spring leaf unfolding advanced by almost two weeks from 1982 to 2011 in Western Europe [3], with an increased risk of incurring possible cold damage of vulnerable plant tissues [4,5,6,7]. Chilling stress (0–15 °C) and freezing stress (below 0 °C) have detrimental effects on plants at both vegetative and reproductive stages, exhibiting symptoms of leaf chlorosis, plant wilting, retarded growth, cell death, and premature senescence [2]. In more detail, cold stress causes membrane rigidification of plant cells, increasing membrane permeability and electrolyte leakage [8], with negative impacts on membrane-associated processes (e.g., photosynthesis and respiration) and homeostasis of reactive oxygen species (ROS) [9]. Thus, plants respond to cold stress by activating acclimation processes, which encompass transcriptional, post-transcriptional, and metabolic changes [9,10]. In particular, plant responses to cold stress include the modulation of cold-stress-related genes, such as C-repeat binding factors (*CBFs*), inducers of CBF expression genes (*ICEs*), and cold-responsive genes (*CORs*), implicated in the ICE-CBF-COR signaling pathway responsible for cold-stress acclimation [11]. In tomato plants (*Solanum lycopersicum* L.), cold-stress response is known to be associated with the upregulation of genes related to calcium-mediated signaling, hormone signaling, response to stimuli, response to stress, ROS homeostasis [12], defense response, response to cold, and response to salt stress [13]. Likewise, cold-stress response included a complex transcriptional reprogramming of genes implicated in antioxidant activities, biosynthesis of sugars and compatible solutes (osmolytes), photosynthetic machinery, phytohormone metabolisms, and transcriptional regulations [14,15,16], indicating an attempted activation of acclimation processes in tomato plants.

Although tomato plants can react to cold stress, they are highly cold-sensitive due to the tropical origin of this crop [1,17]. Most commercial tomato cultivars are sensitive to temperatures below 15 °C, and temperature drops below 12 °C can impair plant growth [18,19]. Thus, tomato plants are frequently grown under greenhouse conditions or plastic-covered tunnels in temperate regions to prevent cold damage [18,19]. Moreover, additional strategies can be implemented to stimulate cold-stress acclimation in tomato plants, such as the application of beneficial microorganisms [18,20,21,22,23,24,25,26,27,28,29,30,31,32] and phytohormones (e.g., abscisic acid (ABA), methyl jasmonate, methyl salicylate, salicylic acid) [1,8,33,34,35]. In particular, cold damage in tomato plants can be mitigated by inoculations with beneficial bacteria isolated from *Arabidopsis thaliana* plants (*Pararhizobium* sp. 44 and *Pararhizobium* sp. 128) [31], Antarctic plants (*Ewingella* sp. S1.OA.A_B6 and *Pseudomonas* sp. S2.OTC.A_B10) [20,21,25], mountain environments (*Pseudomonas* sp. Ps1) [22], Andes Mountains (*Pseudomonas* sp. TmR5a and *Curtobacterium* sp. BmP22c) [23], and soils (*Flavobacterium* sp. OB146 and *Pseudomonas* sp. OB155, OS261, and OS211) [18,24]. Complex microbial communities are known to be associated with plants in cold environments, and they can promote plant growth under cold stress [36]. In particular, cold-tolerant bacterial endophytes belonging to the core taxa of alpine plants (*Duganella*, *Erwinia*, *Pseudomonas*, and *Rhizobium* genera) can mitigate cold stress in strawberry plants [37], suggesting the potential role of alpine bacteria to alleviate cold stress in crops. Beneficial bacteria can activate the acclimation processes of plant hosts and exert positive effects on plant growth at low temperatures, improving nutrient acquisition, photosynthesis efficiency, and biomass production through the modulation of antioxidant responses, hormone signaling, and osmolyte content [38]. The beneficial effects of bacterial inoculation at low temperatures included the decrease in ROS content and the upregulation of genes related to the antioxidant system, cold-stress response (e.g., CBF transcription factors, antioxidant enzymes), photosynthesis, and phytohormone metabolism (e.g., abscisic acid) in tomato shoots [18,28,29,31]. However, transcriptomic regulations stimulated by beneficial bacteria were only partially investigated in tomato plants under cold stress. This study aimed to examine transcriptional processes activated by cold stress in tomato plants following inoculation with cold-tolerant bacteria isolated from alpine plants and to identify tomato genes potentially involved in cold-stress acclimation. The novelty of this study was to inoculate tomato plants with cold-tolerant bacterial endophytes isolated from cold-adapted alpine plants and to investigate transcriptional responses (e.g., genes related to the ICE-CBF-COR signaling pathway) activated by control plants (mock-inoculated) and bacterium-inoculated plants after one day and 14 days of incubation under non-stress (25 ± 2 °C) and cold-stress (10 ± 2 °C) conditions by RNA-Seq analysis.

## 2. Results

### 2.1. Cold-Tolerant Bacterial Isolates Promoted Tomato Growth and Reduced H_2_O_2_ Content Under Cold Stress

**Figure 1:**
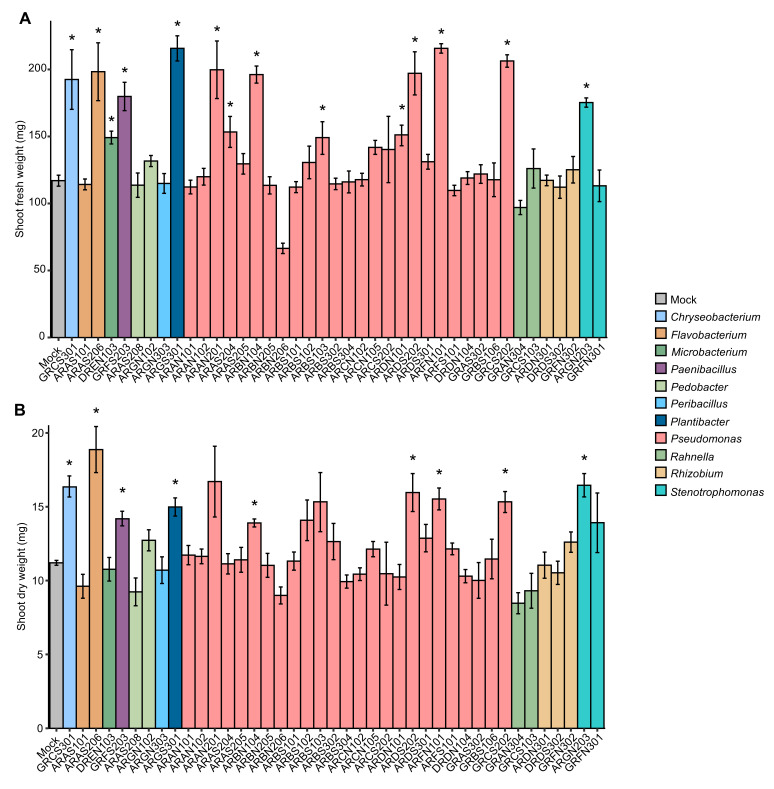
Screening trials of plant growth-promoting bacteria. Tomato plants were treated with MgSO_4_ (mock-inoculated) or inoculated with a bacterial suspension (bacterium-inoculated) of cold-tolerant bacterial isolates specified by the codes and taxonomic annotations (colored legend). Shoot fresh weight (**A**) and shoot dry weight (**B**) were assessed after 28 days of incubation in the growth chamber at 25 ± 2 °C and expressed as mg of each plant. Mean and standard error values of four replicates (pool of four plants) are reported for each inoculation condition. Asterisks indicate significant increases in shoot fresh weight or shoot dry weight in bacterium-inoculated compared to mock-inoculated plants, according to the Mann–Whitney test (*p* ≤ 0.05).

**Figure 2:**
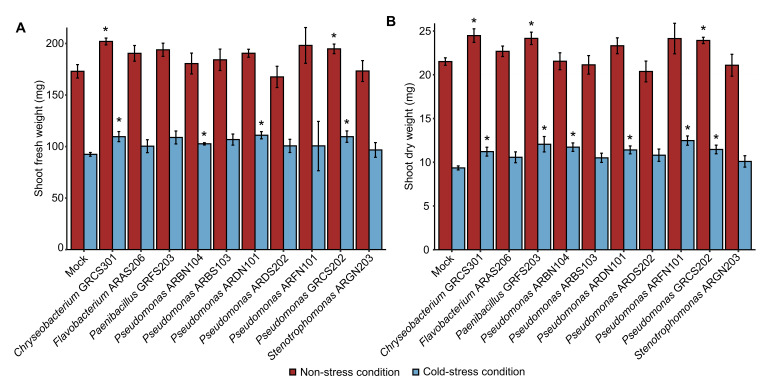
Validation trials of plant growth-promoting bacteria under non-stress and cold-stress conditions. Tomato plants were treated with MgSO_4_ (mock-inoculated) or inoculated with a bacterial suspension (bacterium-inoculated) of cold-tolerant bacterial isolates specified by the codes and taxonomic annotations. Plants were incubated in the growth chamber for 14 days at 25 ± 2 °C, two groups of plants were then obtained, and they were incubated at 25 ± 2 °C (non-stressed plants; red bars) or at 10 ± 2 °C (cold-stressed plants; blue bars) for 14 days. Shoot fresh weight (**A**) and shoot dry weight (**B**) were assessed at the end of the incubation in the growth chamber and expressed as mg of each plant. Mean and standard error values of four replicates (pool of seven plants) are reported for each inoculation condition. Asterisks indicate significant increases in shoot fresh weight or shoot dry weight in bacterium-inoculated compared to mock-inoculated plants, according to the Mann–Whitney test (*p* ≤ 0.05).

The plant growth-promotion activity of 41 cold-tolerant bacteria previously isolated from alpine plants (Table S1) was assessed in the screening trials at 25 ± 2 °C (Figure S1), and the shoot fresh weight was higher in plants inoculated with *Chryseobacterium* sp. GRCS301, *Flavobacterium* sp. ARAS206, *Microbacterium* sp. DREN103, *Paenibacillus* sp. GRFS203, *Plantibacter* sp. ARGS301, *Pseudomonas* sp. ARAN201, ARAS204, ARBN104, ARBS103, ARDN101, ARDS202, ARFN101, GRCS202, and *Stenotrophomonas* sp. ARGN203 compared to mock-inoculated plants after 28 days of incubation (Figure 1A). Likewise, shoot dry weight was higher in plants inoculated with *Chryseobacterium* sp. GRCS301, *Flavobacterium* sp. ARAS206, *Paenibacillus* sp. GRFS203, *Plantibacter* sp. ARGS301, *Pseudomonas* sp. ARBN104, ARDS202, ARFN101, GRCS202, and *Stenotrophomonas* ARGN203 compared to mock-inoculated plants after 28 days of incubation at 25 ± 2 °C in the screening trials (Figure 1B). Ten cold-tolerant bacterial isolates were selected according to plant growth-promotion activity in the screening trials at 25 ± 2 °C and taxonomic annotation (genera representative of the collection), and they were further characterized in the validation trials under non-stress and cold-stress conditions. In the validation trials, mock-inoculated and bacterium-inoculated plants were grown for 14 days in the growth chamber at 25 ± 2 °C, and further incubated at 25 ± 2 °C (non-stressed plants) or at 10 ± 2 °C (cold-stressed plants) for 14 days (Figure S1). The shoot fresh weight was improved by *Chryseobacterium* sp. GRCS301 and *Pseudomonas* sp. GRCS202 inoculation under non-stress conditions, and by *Chryseobacterium* sp. GRCS301, *Pseudomonas* sp. ARBN104, ARDN101, and GRCS202 inoculation under cold-stress conditions in the validation trials (Figure 2A). Moreover, shoot dry weight was higher in plants inoculated with three bacterial isolates (*Chryseobacterium* sp. GRCS301, *Paenibacillus* sp. GRFS203, and *Pseudomonas* sp. GRCS202) and six bacterial isolates (*Chryseobacterium* sp. GRCS301, *Paenibacillus* sp. GRFS203, *Pseudomonas* sp. ARBN104, ARDN101, ARFN101, and GRCS202) compared to mock-inoculated plants under non-stress and cold-stress conditions, respectively (Figure 2B).

**Figure 3:**
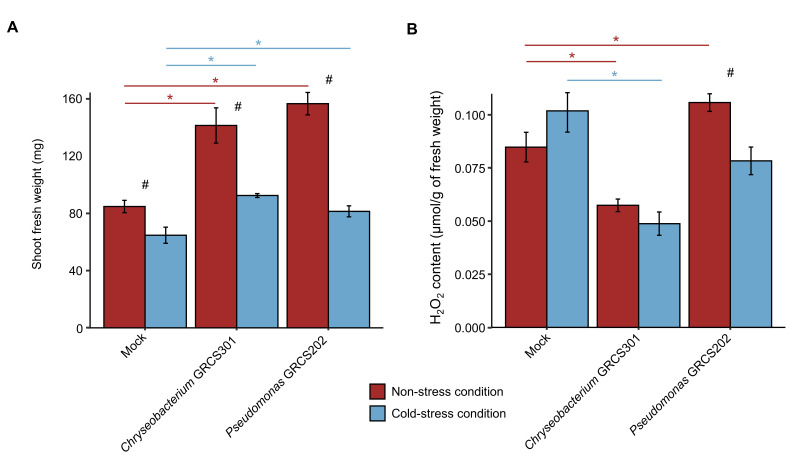
Functional effects of cold-tolerant bacterial isolates on tomato plants under non-stress and cold-stress conditions. Tomato plants were treated with MgSO_4_ (mock-inoculated) or inoculated with *Chryseobacterium* sp. GRCS301 or *Pseudomonas* sp. GRCS202. Plants were incubated in the growth chamber for 14 days at 25 ± 2 °C, two groups of plants were then obtained, and they were incubated at 25 ± 2 °C (non-stressed plants) or at 10 ± 2 °C (cold-stressed plants) for 14 days. Shoot fresh weight (**A**) and H_2_O_2_ content (**B**) were assessed at 14 days and one day after stress exposure (DAS), respectively. Mean and standard error values of three replicates (pool of seven plants) are reported for each inoculation condition, temperature regime, and time point. Asterisks indicate significant differences between bacterium-inoculated and mock-inoculated samples for each temperature regime, according to the Mann–Whitney test (*p* ≤ 0.05). Hashtags indicate significant differences between cold-stressed and non-stressed plants for each inoculation condition, according to the Mann–Whitney test (*p* ≤ 0.05).

*Chryseobacterium* sp. GRCS301 (*Chryseobacterium*) and *Pseudomonas* sp. GRCS202 (*Pseudomonas*) were selected since they belonged to different genera and provided a consistent plant growth-promotion in terms of fresh weight and dry weight in the screening trials and validation trials, and they were further characterized in the functional characterization trials to assess H_2_O_2_ content and transcriptional regulations in tomato shoots under non-stress and cold-stress conditions. In the functional characterization trials, *Chryseobacterium* and *Pseudomonas* confirmed the plant growth-promotion activity under non-stress and cold-stress conditions (Figure 3A). For each inoculum condition, fresh weight was lower in cold-stressed compared to non-stressed plants.

To assess the effect of *Chryseobacterium* and *Pseudomonas* inoculation in cold stress mitigation, H_2_O_2_ content was evaluated in tomato shoots. The content of H_2_O_2_ was lower in *Chryseobacterium*-inculcated compared to mock-inoculated plants under non-stress and cold-stress conditions (Figure 3B). Moreover, the content of H_2_O_2_ tended to decrease in *Pseudomonas*-inoculated compared to mock-inoculated plants under cold-stress conditions, whereas it tended to increase under non-stress conditions. In *Pseudomonas*-inoculated plants, the content of H_2_O_2_ was lower in cold-stressed compared to non-stressed samples. In the re-isolation assay from tomato roots, CFU counts on *Chryseobacterium* medium and *Pseudomonas* medium were higher in bacterium-inoculated compared to mock-inoculated plants under non-stressed and cold-stress conditions (Figure S2).

### 2.2. Cold-Tolerant Bacterial Isolates Affected Transcriptional Responses of Tomato Shoots Under Cold-Stress Conditions

To characterize the tomato response to cold stress and bacterial inoculation, RNA-Seq analysis was carried out for mock-, *Chryseobacterium*-, and *Pseudomonas*-inoculated plants collected in triplicate from non-stress and cold-stress conditions at one and 14 days after stress exposure (DAS), and sequences were deposited at NCBI (BioProject number PRJNA1079540). From 45.70% to 87.87% of filtered paired-end reads aligned to the tomato reference genome (*Solanum lycopersicum* ITAG5.0), and 21,698 genes resulted as active [reads per million mapping to the gene (RPM) > 1 in at least one sample; Table S2]. Principal component analysis (PCA) revealed clustering of replicates and separation of samples according to the temperature regime (first component) and time point (second component; Figure S3). The accuracy of RNA-Seq results was validated by quantitative real-time PCR (qPCR) using seven genes selected as known markers of tomato response to cold stress [14,28,39], such as 9-cis-epoxycarotenoid dioxygenase (*NCED5*), C-repeat/DRE binding factor 1 (*CBF1*), chalcone synthase (*CHS2*), elongated hypocotyl 5 transcriptional factor (*HY5*), flavonoid-3’-hydroxylase (*F3’5’H*), glutathione-S-transferase enzyme (*GST*), and sucrose synthase (*SUS*; Table S3). A positive correlation (R^2^ of 0.84) between Log_2_-transformed fold change (LFC) values assessed by RNA-Seq and qPCR was obtained (Figure S4).

**Figure 4:**
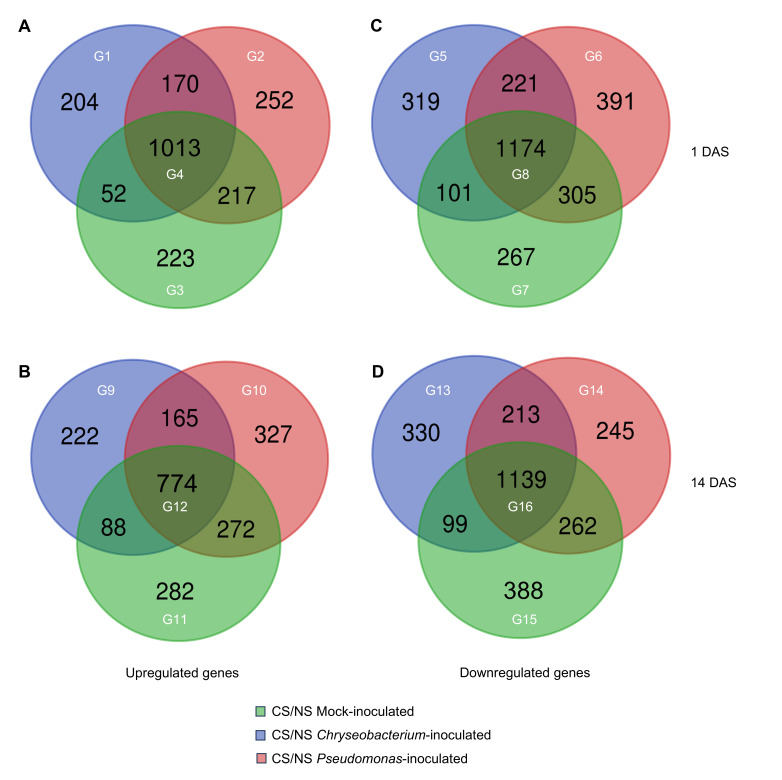
Differential expression analysis results. Tomato plants were treated with MgSO_4_ (mock-inoculated) or inoculated with *Chryseobacterium* sp. GRCS301 (*Chryseobacterium*-inoculated) or *Pseudomonas* sp. GRCS202 (*Pseudomonas*-inoculated), and shoot samples were collected at one day (1 DAS) and 14 days (14 DAS) after stress exposure (DAS) from plants incubated at 25 ± 2 °C (non-stress condition; NS) or at 10 ± 2 °C (cold-stress condition; CS). Venn diagrams summarize the number (black numbers) of upregulated (**A**,**B**) and downregulated (**C**,**D**) differentially expressed genes (DEGs) at 1 DAS and 14 DAS in the pairwise comparisons between CS and NS samples for each inoculation condition specified by the color legend. Sixteen groups (G; white numbers) of genes modulated in CS samples compared to NS samples in all inoculation conditions (G4, G8, G12, and G16) or exclusively in mock- (G3, G7, G11, and G15), *Chryseobacterium*- (G1, G5, G9, and G13), or *Pseudomonas*- (G2, G6, G10, and G14) inoculated plants were selected for further analyses.

A total of 4909 and 4806 differentially expressed genes (DEGs) were found at 1 DAS and 14 DAS, respectively, by differential expression analysis imposing a false discovery rate (FDR) lower than 0.05 (adjusted *p*-value ≤ 0.05) and a Log_2_-transformed fold change (LFC) higher than 1 or lower than −1 in the pairwise comparisons between the cold-stressed and non-stress condition of mock-inoculated plants (cold-stressed mock-inoculated vs. non-stressed mock-inoculated), *Crhyseobacterium*-inoculated plants (cold-stressed *Chryseobacterium*-inoculated vs. non-stressed *Chryseobacterium*-inoculated), and *Pseudomonas*-inoculated plants (cold-stressed Pseudomonas-inoculated vs. non-stressed *Pseudomonas*-inoculated; Table S4). In particular, 2131 and 2130 DEGs were upregulated at 1 DAS and 14 DAS, whereas 2778 and 2676 DEGs were downregulated at 1 DAS and 14 DAS, respectively. For each time point, upregulated and downregulated DEGs were grouped in those modulated by cold stress in all inoculation conditions or exclusively modulated in mock-, *Chryseobacterium*-, or *Pseudomonas*-inoculated plants, to highlight possible common and specific responses to cold stress (Figure 4). A large fraction of DEGs was modulated by cold stress in all inoculation conditions (groups G4, G8, G12, and G16), as a possible consistent response of tomato shoots to cold stress. Moreover, *Chryseobacterium* inoculation (groups G1, G5, G9, and G13) and *Pseudomonas* inoculation (groups G2, G6, G10, and G14) highlight specific transcriptional responses to cold stress compared to mock-inoculated plants (groups G3, G7, G11, and G15) at both time points. In particular, 204 and 252 tomato genes were upregulated by cold stress exclusively in *Chryseobacterium*- and *Pseudomonas*-inoculated plants, respectively, while 223 genes were upregulated by cold stress exclusively in mock-inoculated plants at 1 DAS. At 14 DAS, 282, 222, and 327 genes were upregulated by cold stress exclusively in mock-, *Chryseobacterium*-, and *Pseudomonas*-inoculated plants, respectively. Moreover, genes downregulated by cold stress were 267 and 388 in mock-inoculated plants, 319 and 330 in *Chryseobacterium*-inoculated plants, and 391 and 245 in *Pseudomonas*-inoculated plants at 1 and 14 DAS, respectively.

**Figure 5:**
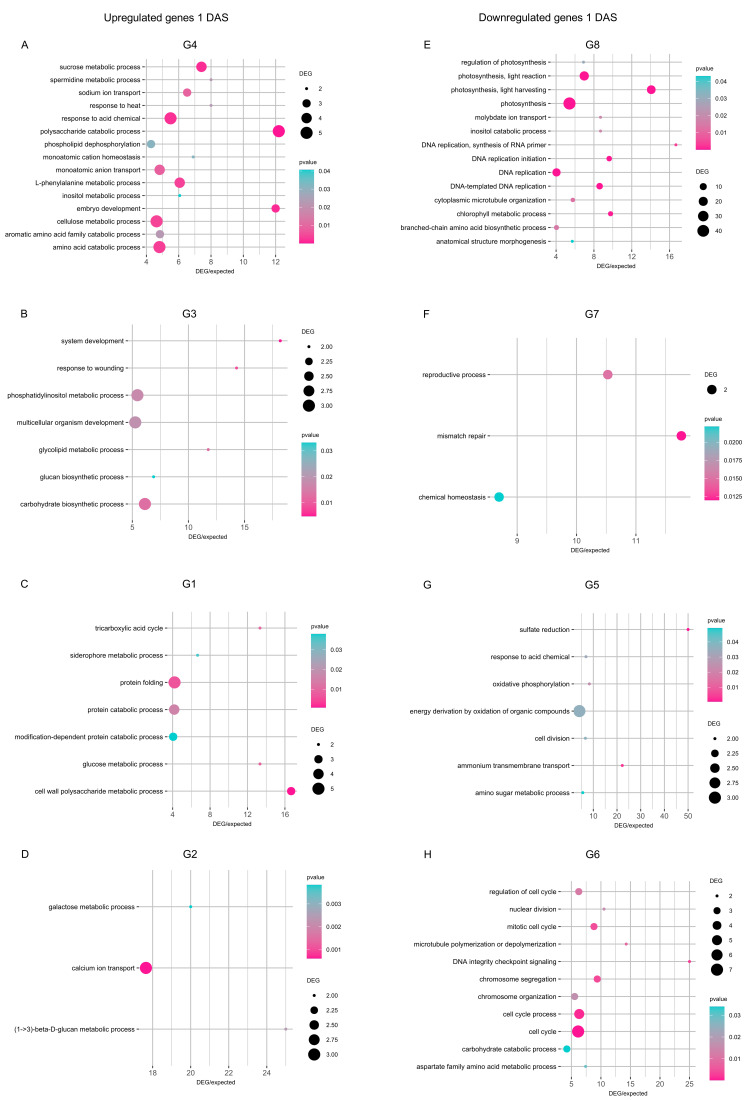
Enriched functional categories of genes modulated one day after stress exposure (DAS). Gene ontology (GO) enrichment analysis was carried out for genes upregulated (**A**–**D**) and downregulated (**E**–**H**) in the pairwise comparison between the cold-stressed and non-stressed samples in all inoculation conditions (**A**,**E**) or exclusively in mock-inoculated plants (**B**,**F**), *Chryseobacterium*-inoculated plants (**C**,**G**), or *Pseudomonas*-inoculated plants (**D**,**H**) at 1 DAS. The number of DEGs is reported according to the dimension scale, and the significance of Fisher’s test (*p* ≤ 0.05) is reported according to the color legend for each functional category, with a ratio between DEGs and expected genes higher than five. The group (G) numbers of DEGs are referred to Venn diagrams of Figure 4, such as genes modulated in cold-stressed samples (CS) compared to non-stressed samples (NS) in all inoculation conditions (G4, G8, G12, and G16) or exclusively in mock- (G3, G7, G11, and G15), *Chryseobacterium*- (G1, G5, G9, and G13), or *Pseudomonas*- (G2, G6, G10, and G14) inoculated plants were selected for further analyses.

DEGs upregulated by cold stress in all inoculation conditions at 1 DAS (group G4) revealed the enrichment mainly of amino acid catabolic process, phenylalanine metabolic process, polysaccharide catabolic process, response to acid chemical, and sucrose metabolic process (*p* ≤ 0.05 and DEG/expected genes ratio ≥ 5; Figure 5A). DEGs upregulated by cold stress exclusively in mock-inoculated plants at 1 DAS (group G3) revealed the enrichment of carbohydrate biosynthetic process, multicellular organism development, and phosphatidylinositol metabolic process (Figure 5B). Functional categories of cell wall polysaccharide metabolic process, protein catabolic process, and protein folding were enriched in DEGs exclusively upregulated in *Chryseobacterium*-inoculated plants (group G1; Figure 5C), while functional categories of calcium ion transport, glucan metabolic process, and galactose metabolic process were enriched in DEGs exclusively upregulated in *Pseudomonas*-inoculated plants at 1 DAS (group G2; Figure 5D). DEGs downregulated by cold stress at 1 DAS showed the enrichment of DNA replication and photosynthesis in all inoculation conditions (group G8; Figure 5E), chemical homeostasis and reproductive process in mock-inoculated plants (group G7; Figure 5F), energy derivation by oxidation of organic compounds in *Chryseobacterium*-inoculated plants (group G5; Figure 5G), and cell cycle-related processes in *Pseudomonas*-inoculated plants (Figure 5H).

**Figure 6:**
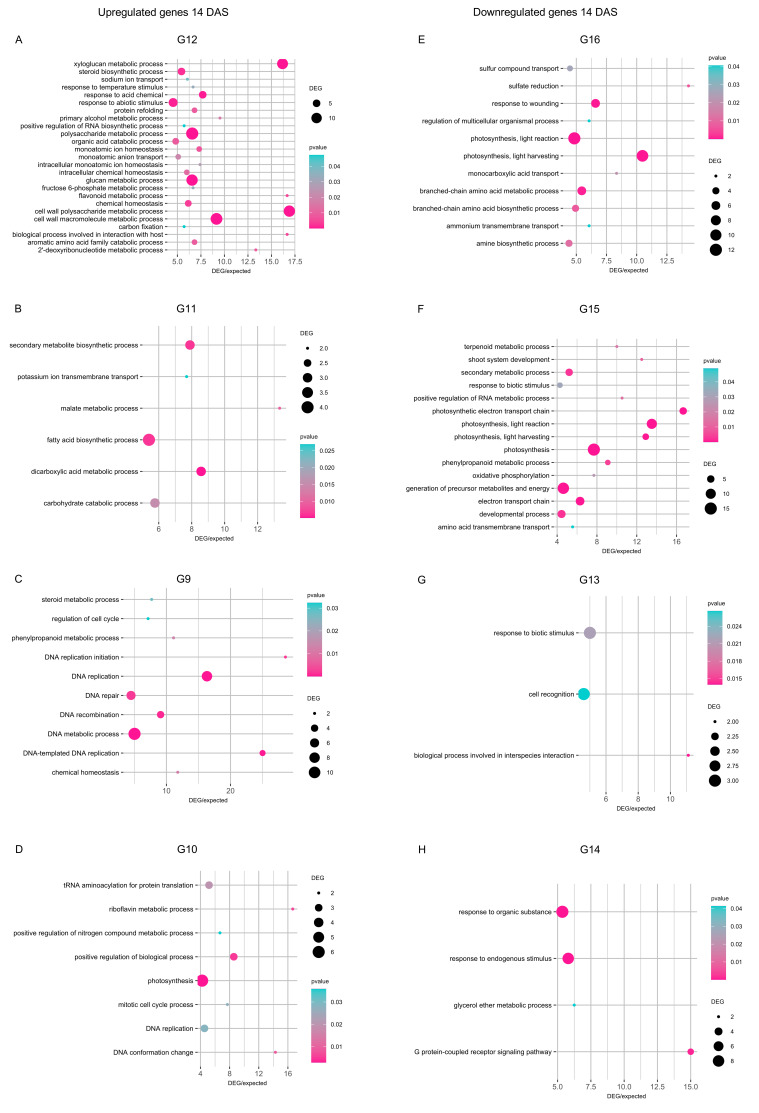
Enriched functional categories of genes modulated 14 days after stress exposure (DAS). Gene ontology (GO) enrichment analysis was carried out for genes upregulated (**A**–**D**) and downregulated (**E**–**H**) in the pairwise comparison between the cold-stressed and non-stressed samples in all inoculation conditions (**A**,**E**) or exclusively in mock-inoculated plants (**B**,**F**), *Chryseobacterium*-inoculated plants (**C**,**G**), or *Pseudomonas*-inoculated plants (**D**,**H**) at 14 DAS. The number of DEGs is reported according to the dimension scale, and the significance of Fisher’s test (*p* ≤ 0.05) is reported according to the color legend for each functional category, with a ratio between DEGs and expected genes higher than five. The group (G) numbers of DEGs are referred to Venn diagrams of Figure 4, such as genes modulated in cold-stressed samples (CS) compared to non-stressed samples (NS) in all inoculation conditions (G4, G8, G12, and G16) or exclusively in mock- (G3, G7, G11, and G15), *Chryseobacterium*- (G1, G5, G9, and G13), or *Pseudomonas*- (G2, G6, G10, and G14) inoculated plants were selected for further analyses.

At 14 DAS, DEGs upregulated by cold stress in all inoculation conditions (group G12) revealed the enrichment mainly of cell wall macromolecule metabolic process, glucan and xyloglucan metabolic processes, polysaccharide metabolic process, and response to abiotic stimulus (*p* ≤ 0.05 and DEG/expected genes ratio ≥ 5; Figure 6A). Moreover, DEGs upregulated by cold stress exclusively in mock-inoculated plants at 14 DAS (group G11) revealed the enrichment of carbohydrate catabolic process, dicarboxylic acid metabolic process, fatty acid biosynthetic process, and secondary metabolite biosynthetic process (Figure 6B). Functional categories of DNA metabolic process and DNA replication were enriched in DEGs exclusively upregulated in *Chryseobacterium*-inoculated plants (group G9; Figure 6C), while photosynthesis, protein translation, and regulation of biological process were enriched in DEGs exclusively upregulated in *Pseudomonas*-inoculated plants at 14 DAS (group G10; Figure 6D). DEGs downregulated by cold stress at 14 DAS showed the enrichment of photosynthesis, response to wounding, and amino acid metabolic processes in all inoculation conditions (group G16; Figure 6E), photosynthesis and generation of precursor metabolites and energy in mock-inoculated plants (group G15; Figure 6F), cell recognition and response to biotic stimulus in *Chryseobacterium*-inoculated plants (group G13; Figure 6G), response to endogenous stimulus and response to organic substance in *Pseudomonas*-inoculated plants (group G14; Figure 6H). In addition to stress-related pathways, DEGs revealed the enrichment of more general functional categories (e.g., embryo development, ion transport, response to acid chemical, and sulfate reduction), as possible complex transcriptional reprogramming to bacterial inoculation that involved pathways not limited to stress tolerance.

**Figure 7:**
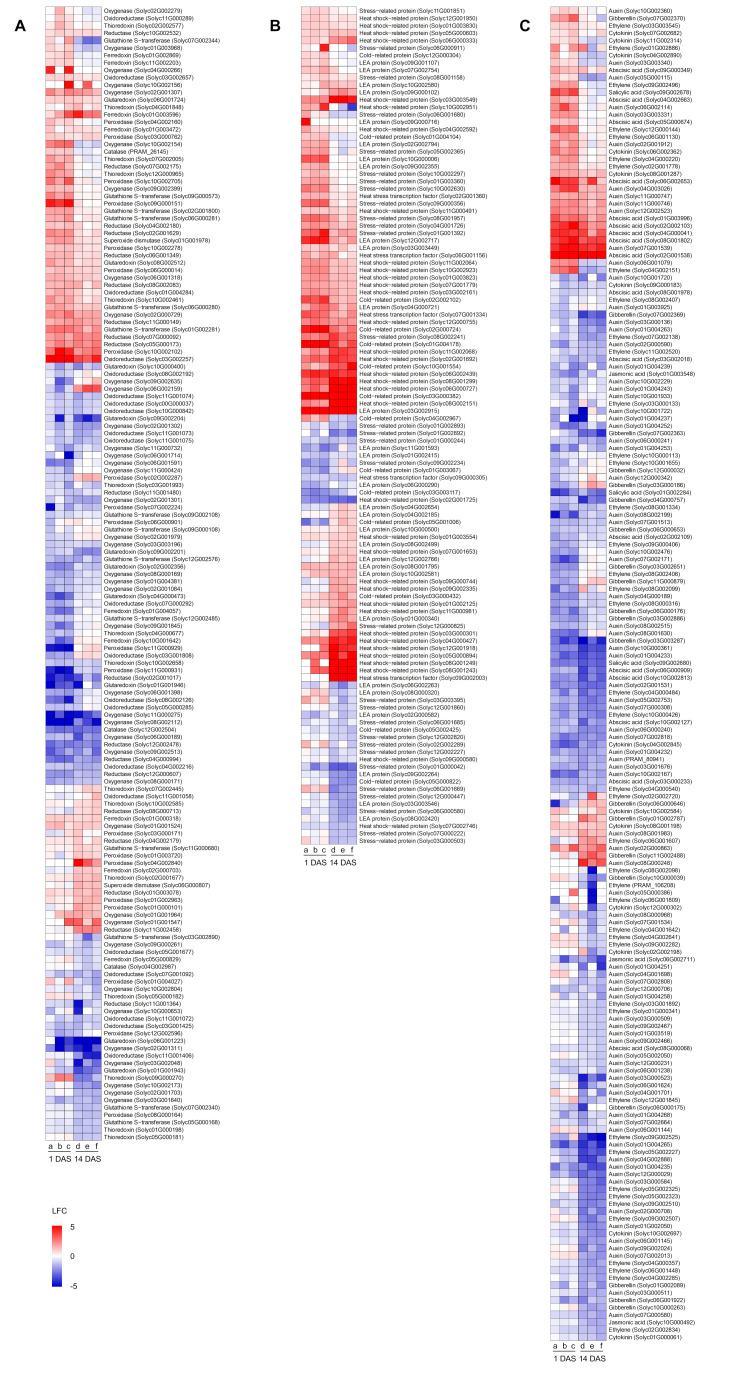
Heatmaps of differentially expressed genes (DEGs) modulated by cold stress and belonging to functional categories possibly involved in cold stress mitigation, such as oxidative stress (**A**), stress response (**B**), and hormonal signaling (**C**). Log2-transformed fold change values (color legend; LFC) of tomato DEGs are reported for each pairwise comparison between cold-stress (CS) and non-stress (NS) samples of mock-inoculated plants (columns a and d), *Chryseobacterium*-inoculated plants (columns b and e), and *Pseudomonas*-inoculated plants (columns c and f) collected at one day and 14 days after stress exposure (DAS). DEGs were assigned to functional categories according to protein descriptions (Table S4) and sorted according to hierarchical clustering on expression profiles. Abbreviations: LEA, late embryogenesis abundant.

In order to better highlight genes possibly involved in cold stress mitigation, heatmaps summarizing the expression profiles DEGs related to oxidative stress, stress response, and hormonal signaling were obtained (Figure 7 and Table S4). Several DEGs possibly related to oxidative stress response were modulated by cold stress in mock-, *Chryseobacterium*-, or *Pseudomonas*-inoculated plants, such as three catalase (Solyc04G002987, Solyc12G002504, and PRAM_26145), nine ferredoxin (Solyc01G000318, Solyc01G002869, Solyc01G003472, Solyc01G003596, Solyc01G004057, Solyc02G000703, Solyc05G000829, Solyc10G001642, and Solyc11G002203), ten glutaredoxin (Solyc01G001943, Solyc01G001946, Solyc02G002356, Solyc04G000473, Solyc06G001223, Solyc06G001724, Solyc08G002512, Solyc09G002201, Solyc09G002204, and Solyc10G000400), 14 glutathione S-transferase (Solyc01G002281, Solyc02G001800, Solyc03G002890, Solyc05G000168, Solyc06G000280, Solyc06G000281, Solyc07G002340, Solyc07G002344, Solyc09G000108, Solyc09G000573, Solyc09G002108, Solyc11G000680, Solyc12G002485, and Solyc12G002576), 21 oxidoreductase, 40 oxygenase, 20 peroxidase, 19 reductase, two superoxide dismutase (Solyc01G001978 and Solyc06G000807), and 15 thioredoxin-related genes (Figure 7A and Table S4). Transcriptional reprogramming of mock-, *Chryseobacterium*-, or *Pseudomonas*-inoculated plants to cold stress included the modulation of genes possibly related to stress response, such as 14 cold-related proteins (Solyc01G003067, Solyc01G004104, Solyc01G004178, Solyc02G000724, Solyc02G002102, Solyc03G000382, Solyc03G000432, Solyc03G003117, Solyc04G002967, Solyc05G000822, Solyc05G001006, Solyc05G002425, Solyc10G001554, and Solyc12G000304), five heat stress transcription factors (Solyc02G001360, Solyc06G001156, Solyc07G001334, Solyc09G000305, and Solyc09G002003), 35 heat shock-related proteins, 29 late embryogenesis abundant proteins, and 30 stress-related proteins (Figure 7B and Table S4). Moreover, genes related to hormonal signaling were modulated by cold stress in mock-, *Chryseobacterium*-, or *Pseudomonas*-inoculated plants, such as 81 auxin-, 13 cytokinin-, 43 ethylene-, 20 gibberellin-, 17 ABA- (Solyc01G003996, Solyc02G001538, Solyc02G002103, Solyc02G002109, Solyc03G000233, Solyc03G002018, Solyc04G000041, Solyc04G002663, Solyc05G000674, Solyc06G000909, Solyc06G002653, Solyc08G000068, Solyc08G001802, Solyc08G001978, Solyc09G000349, Solyc10G002127, and Solyc10G002813), four jasmonic acid- (Solyc01G003548, Solyc05G002124, Solyc06G002711, and Solyc10G000492), and three salicylic acid- (Solyc01G002284, Solyc09G002678, Solyc09G002680) related genes (Figure 7C and Table S4).

## 3. Discussion

Although tomato plants try to activate the acclimation processes against cold stress [11,14,15,16], they remain cold-sensitive [1,17]. In this study, we found that cold-tolerant bacteria isolated from alpine plants [37] can promote shoot growth in tomato plants under non-stress conditions. In particular, four (*Chryseobacterium* sp. GRCS301, *Pseudomonas* sp. ARBN104, ARDN101, and GRCS202) and six (*Chryseobacterium* sp. GRCS301, *Paenibacillus* sp. GRFS203, *Pseudomonas* sp. ARBN104, ARDN101, ARFN101, and GRCS202) cold-tolerant bacteria can promote fresh weight and shoot dry weight under cold-stress conditions, respectively, suggesting their contribution to cold-stress mitigation. Slight differences in plant growth-promotion effects were observed in the screening trials and validation trials, possibly due to variations in root colonization levels under non-sterilized growth conditions. However, *Chryseobacterium* sp. GRCS301 and *Pseudomonas* sp. GRCS202 showed consistent plant growth-promoting effects in terms of fresh weight and dry weight in the screening trials and validation trials, and they were further used in the functional characterization trials to analyze their effects on H_2_O_2_ content and transcriptional responses of tomato plants under cold stress.

Inoculation with *Chryseobacterium* and *Pseudomonas* lowered H_2_O_2_ content in cold-stressed plants, suggesting enhanced acclimation compared to mock-inoculated plants. Gene expression results showed enhanced modulation of genes encoding antioxidant enzymes (e.g., glutathione S-transferases, oxidoreductases, oxygenases, reductases, and thioredoxin-related proteins) in *Chryseobacterium*- or *Pseudomonas*-inoculated plants, as a possible increase in antioxidant activity in bacterium-inoculated plants. Likewise, the beneficial effects of bacterial inoculations under cold stress were previously associated with the decrease in ROS content and activation of antioxidant machinery in tomato plants [18,24,28,29,30,31]. Thus, cold-tolerant bacteria previously showed plant growth-promoting activity and stress mitigation in tomato plants at low temperatures, such as a tomato endophyte (*Pseudomonas* sp. TPs-04) [30], plant growth-promoting bacteria (*B. cereus* AR156, *B. subtilis* SM21, *Serratia* sp. XY21, and *Streptomyces* sp. TOR3209) [28,29], soil bacteria (*Flavobacterium* sp. OB146 and *Pseudomonas* sp. OB155, OS261, and OS211) [18,24], mountain isolates (*Pseudomonas* sp. Ps1, *Pseudomonas* sp. TmR5a, and *Curtobacterium* sp. BmP22c) [22,23], Arabidopsis-associated bacteria (*Pararhizobium* sp. 44 and *Pararhizobium* sp. 128) [31], and Antarctic bacteria (*Ewingella* sp. S1.OA.A_B6 and *Pseudomonas* sp. S2.OTC.A_B10) [20,21,25], indicating beneficial effects under cold-stress conditions according to bacterial taxa and isolation environments.

Beneficial bacteria can contribute to cold-stress mitigation in crop plants through activation of acclimation processes [38]. In this study, transcriptional responses of tomato plants to cold stress were affected by *Chryseobacterium* and *Pseudomonas* inoculation. In particular, mock-inoculated plants responded to cold stress mainly with the downregulation of genes related to the generation of energy, photosynthesis, and reproductive processes, indicating negative impacts of cold stress on growth-related processes. On the other hand, cold-stress response involved the activation of pathways mainly related to DNA replication, polysaccharide metabolism, and protein metabolism in *Chryseobacterium*-inoculated plants, or galactose metabolism and photosynthesis in *Pseudomonas*-inoculated plants, suggesting efficient activation of acclimation processes in bacterium-inoculated plants against cold stress. Likewise, genes related to photosynthetic processes, hormonal signaling (e.g., ABA), stress-related metabolisms, carbohydrate metabolisms, and protein metabolisms were upregulated by *Streptomyces* sp. TOR3209 inoculation in tomato leaves exposed to cold stress [28], indicating overlapping pathways stimulated by different beneficial bacteria in tomato plants against cold stress. Here, we found that genes related to hormonal signaling were modulated by cold stress mainly in *Chryseobacterium*- and *Pseudomonas*-inoculated plants, such as genes related to metabolism and signaling of ABA, auxin, cytokinin, ethylene, gibberellin, and jasmonic acid. Among them, genes with regulatory roles in hormonal pathways were found, such as ABA receptor *PYL* genes (Solyc03G000233, Solyc03G002018, Solyc06G000909, Solyc08G001978, Solyc10G002127, Solyc10G002813, Solyc09G002282, Solyc09G002525, Solyc10G000113, Solyc10G000426, Solyc10G001655, Solyc11G002520, andSolyc12G001845) that were previously associated with tomato response to cold stress through the PYR/PYL-PP2C-SnRK2 signaling cascade [40] and the transcription factor ABSCISIC ACID INSENSITIVE 5 (ABI5) responsible for the transcriptional regulation of late embryogenesis abundant proteins [40]. Ethylene and salicylic acid plays a positive role in cold stress tolerance in tomato [40,41], and ethylene-responsive transcription factor (ERF) genes (Solyc02G002834, Solyc03G000133, Solyc03G001892, Solyc03G003545, Solyc04G000484, Solyc04G000540, Solyc04G001642, Solyc04G002151, Solyc04G002285, Solyc04G002641, Solyc05G002227, Solyc05G002323, Solyc05G002325, Solyc06G001448, Solyc06G001607, Solyc06G001809, Solyc07G002138, Solyc08G002098, Solyc08G002099, Solyc09G002282, Solyc09G002525, Solyc10G000113, Solyc10G000426, Solyc10G001655, Solyc11G002520, and Solyc12G001845), 1-aminocyclopropane-1-carboxylate synthase genes (Solyc01G002886, Solyc02G001778, Solyc02G002720, Solyc08G000316, Solyc08G002406, and Solyc08G002407), 1-aminocyclopropane-1-carboxylate oxidase genes (Solyc04G000220, Solyc04G000357, Solyc09G000406, Solyc09G002496, Solyc09G002507, Solyc09G002510, and Solyc12G000144), and salicylic acid biosynthesis genes (Solyc01G002284, Solyc09G002678, and Solyc09G002680) were modulated by cold stress mainly in *Chryseobacterium*- and *Pseudomonas*-inoculated plants, possibly to promote acclimation. Plant responses to low temperatures include complex hormonal regulations, and exogenous phytohormone applications can mitigate cold stress in tomato plants, such as ABA, methyl jasmonate, methyl salicylate, and salicylic acid [1,8,33,34,35]. Likewise, chemical treatments (e.g., 5-aminolevulinic acid and coronatine) can enhance chilling tolerance of tomato plants by modulating genes related to hormonal signaling [16,42], corroborating that cold-stress acclimation is mediated by hormonal regulations.

Genes encoding cold-related proteins were mainly upregulated in *Chryseobacterium*- and *Pseudomonas*-inoculated plants, such as CBF transcription factors (Solyc03G003117, Solyc03G000432, and Solyc05G001006), heat shock-related proteins, late embryogenesis abundant proteins, and oxidative stress-related proteins. Likewise, bacterial inoculations were previously associated with the upregulation of CBF transcription factors to modulate cold-related genes [24,29,31], suggesting that inoculations with beneficial bacteria can reinforce the activation of ICE-CBF-COR signaling pathways in tomato plants, enhancing the cold-stress acclimation capacity. Dehydrins [15] and late embryogenesis abundant proteins [43] can accumulate in response to water deficit induced by low temperature, and *Streptomyces* sp. TOR3209 inoculation can upregulate a dehydrin gene (*TAS14*) to mitigate cold stress in tomato plants [28]. Moreover, the expression of heat shock proteins is known to be upregulated by cold stress in cold-tolerant tomato genotypes [12] or by methyl jasmonate and methyl salicylate treatments against cold stress in tomato fruits [44], possibly contributing to proper protein folding and stress acclimation. However, a large fraction of DEGs (1013 upregulated and 1174 downregulated genes at 1 DAS, 774 upregulated and 1139 downregulated genes at 14 DAS) were modulated by cold stress in all inoculation conditions, indicating that consistent transcriptional responses to cold stress partially occurred in mock- and bacterium-inoculated plants. These consistent responses involved the activation of pathways mainly related to cell wall metabolism, amino acid metabolism, response to stimuli, and sugar metabolism, as well as the downregulation of genes associated with photosynthesis-related processes. The attempted activation of acclimation processes (e.g., response to cold, response to stimuli, response to stress, and ROS homeostasis) and rearrangement of photosynthesis-related processes were previously observed in tomato plants [12,13,14,15]. The reprogramming of sugar metabolism can accumulate osmolytes (e.g., trehalose, raffinose, and polyamines) with cryoprotective properties [15]. In particular, bacterial inoculation of *Pararhizobium* sp. can stimulate polyamine biosynthesis through gene modulation [31], indicating the activation of multiple pathways by beneficial bacteria against cold stress.

These results indicate that tomato inoculation with *Chryseobacterium* sp. GRCS301 and *Pseudomonas* sp. GRCS202 can mitigate cold stress in tomato plants (e.g., promoting shoot growth and decreasing ROS content) by upregulating the expression of genes involved in cold stress-related pathways (e.g., ROS homeostasis, cold-stress response, and hormonal signaling). Although sterilized soils and controlled conditions were applied in our study, the possible contribution of additional factors (e.g., microbial contamination, pathogen infection, and other abiotic or biotic stresses) to tomato gene modulation observed in the RNA-Seq analysis cannot be completely excluded. Thus, the involvement of tomato genes in cold stress acclimation should be further validated by qPCR analyses in additional experiments using different tomato genotypes. Further experiments are also required to analyze the enzymatic activity of antioxidant enzymes at different time points in tomato samples and to verify if hormonal changes are implicated upstream of the transcriptional regulations observed. Moreover, validations under field conditions are required to confirm the effects of bacterial inoculants against oxidative stress caused by low temperatures, and to compare their efficacy with other plant protection strategies (e.g., hormonal treatments) in agronomic contexts.

## 4. Materials and Methods

### 4.1. Bacterial Isolates and Inoculum Preparation

Cold-tolerant bacterial endophytes were previously isolated from the roots of three alpine *Rosaceae* plants (*Alchemilla* sp., *Dryas octopetala,* and *Geum montanum*) [37]. Bacterial isolates were grown in agar (R2A, Millipore, Merck, Rahway, NJ, USA) at 25 ± 1 °C. The 16S rRNA gene sequence of each bacterial isolate was previously obtained [37] (accession numbers in Table S1), and it was analyzed by nucleotide alignment (BLASTn search) against the 16S rRNA database of the National Center for Biotechnology Information (NCBI). The first ten best hits of the alignments were used to verify the non-pathogenicity for humans and crop plants, according to the literature search, and a total of 41 isolates were selected for functional characterization in tomato plants (Table S1).

To prepare the bacterial suspension for plant inoculation, each isolate was grown overnight (18 h) in liquid Reasoner’s 2 Broth (R2B, Microbiol Diagnostici, Cagliari, Italy) at 25 ± 1 °C under orbital shaking at 180 rpm. Bacterial cells were collected by centrifugation (3500× *g* for 10 min) and washed three times with sterile 10 mM MgSO_4_. According to the conversion of optical density (OD) at 600 nm (OD_600_), the bacterial suspension was adjusted to 1.0 × 10^8^ colony-forming units (CFU) per unit of volume (CFU mL^−1^) using a spectrophotometer (Ultrospec 3100; GE Healthcare, Chicago, IL, USA). For the two cold-tolerant bacterial isolates used in the functional characterization experiments, the OD_600_ 0.1 corresponded to 8.9 × 10^7^ CFU mL^−1^ for *Chryseobacterium* sp. GRCS301 (*Chryseobacterium*) and 8.7 × 10^7^ CFU mL^−1^ for *Pseudomonas* sp. GRCS202 (*Pseudomonas*).

### 4.2. Plant Material and Growth Conditions

Seeds of *S. lycopersicum* L. cultivar Moneymaker (Justseed, Wrexham, UK) were surface disinfected by incubation in 70% ethanol for 1 min, 2% sodium hypochlorite for 5 min, and 70% ethanol for 1 min, followed by three washes with sterile water (3 min each) in a 50 mL tube under moderate shaking [25]. Surface-disinfected seeds were placed into a 100 cm^2^-square dish (100 seeds per dish; Sarstedt, Nümbrecht, Germany) containing 10 g L^−1^ water agar (0.8% Agar Technical, Oxoid, Basingstoke, Hampshire, UK). Plates were incubated for two days at 25 ± 2 °C in the dark to allow seed germination. Germinated seeds with the same root length (2 mm) were transplanted in pots (45 cm^3^) containing a twice-sterilized mixture [(1:2) peat:sand (1:2) mixture] of peat (Semina 80 Tecno Grow, Tercomposti, Calvisano, Italy) and silica sand (Type 519, Bacchi, Boretto, Italy). Seedlings were incubated in a growth chamber (Ekochl 700, Angelantoni Life Science, Milano, Italy) at 25 ± 2 °C with a 14:10 light–dark photoperiod (photon flux density of 0.033 mmol s^−1^ m^−2^). Plants were treated with 1.5 ml of sterile 10 mM MgSO_4_ (mock-inoculated) or inoculated with 1.5 ml of the suspension (1.0 × 10^8^ CFU mL^−1^) of the respective cold-tolerant bacterial isolate (bacterium-inoculated) in a randomized block design. Treatments were applied at the root collar every seven days starting from the transfer into the soil–sand mixture, for a total of four applications to each plant (Figure S1).

To select plant growth-promoting bacteria, screening trials with the 41 cold-tolerant bacterial isolates were carried out, and plants were incubated in the growth chamber for 28 days at 25 ± 2 °C with a 14:10 light–dark photoperiod. The plant growth-promotion activity of the best-performing bacterial isolates was further analyzed under non-stress and cold-stress conditions in the validation trials and functional characterization trials. In the validation trials and functional characterization trials, mock-inoculated and bacterium-inoculated plants were grown for 14 days in the growth chamber at 25 ± 2 °C with a 14:10 light–dark photoperiod, two groups of plants were then obtained by random selection within each inoculation condition, and they were incubated at 25 ± 2 °C in the growth chamber (non-stressed plants) or at 10 ± 2 °C in another growth chamber (cold-stressed plants) for 14 days with a 14:10 light–dark photoperiod (Figure S1). Four replicates (screening trials and validation trials) or three replicates (functional characterization trials) were analyzed for each inoculation condition and temperature regime, and each replicate consisted of a pool of four plants (screening trials) or seven plants (validation trials and functional characterization trials). The number of plants of each experiment was set according to the available space in the growth chambers, and a larger number of plants per replicate was used in the validation trials and functional characterization trials compared to screening trials to obtain sufficient shoot material for the subsequent analyses (H_2_O_2_ content and RNA Extraction).

### 4.3. Sample Collection and Assessment of Fresh Weight and Dry Weight

In the screening trials, validation trials, and functional characterization trials, tomato shoots of each replicate (pool of at least four plants) were collected at the end of the incubation in the growth chamber. The fresh weight was immediately assessed using a precision balance (E42, Gibertini, Milano, Italy) and expressed as mg of each plant. In the screening trials and validation trials, each shoot sample was incubated at 60 °C for 48 h, and the dry weight of each replicate was assessed with the precision balance and expressed as mg of each plant.

In the functional characterization trials, tomato shoots of mock-, *Chryseobacterium*-, and *Pseudomonas*-inoculated plants were collected at one day and 14 DAS from non-stressed and cold-stressed plants. These time points were chosen to analyze the early [14] and late [39] transcriptional response of tomato plants to cold stress. Moreover, 1 DAS was chosen to assess the content of H_2_O_2_ in response to cold stress [35]. Each replicate of shoot samples was immediately frozen in liquid nitrogen and stored at −80 °C. Shoot samples were ground to a fine powder using a mixer mill disruptor (MM 400, Retsch, Haan, Germany) at 25 Hz for 60 s with 2 mL-tubes and 6 mm-beads refrigerated in liquid nitrogen, and the resulting powder was stored at −80 °C until further analysis. Three replicates (pool of seven plants) were obtained for each inoculation condition (mock-, *Chryseobacterium*-, and *Pseudomonas*-inoculated plants), temperature regime (non-stressed and cold-stressed plants), and time point (1 DAS and 14 DAS).

### 4.4. Bacterial Re-Isolation from Tomato Roots

In the functional characterization trials, tomato roots from each replicate were collected at the end of the incubation in the growth chamber, and rhizosphere samples were obtained as previously described [45] with slight modifications. Briefly, large soil aggregates were removed by shaking the roots, and the fresh weight of tomato roots was assessed using the precision balance. Root samples were transferred to a 15 ml tube containing 2.5 mL sterile 10 mM MgSO_4_ and 0.01% Tween 20, and incubated for 20 min under orbital shaking at 180 rpm. Each suspension was serially diluted, and aliquots (10 μL) were plated in triplicate on a semi-selective R2A medium containing 2.5 μg mL^−1^ cycloheximide, 2 μg mL^−1^ erythromycin, 10 μg mL^−1^ gentamycin, and 5 μg mL^−1^ kanamycin to isolate *Chryseobacterium* spp. (*Chryseobacterium* medium), or semi-selective R2A medium containing 2.5 μg mL^−1^ cycloheximide, 30 μg mL^−1^ chloramphenicol, and 5 μg mL^−1^ tobramycin sulfate to isolate *Pseudomonas* spp. (*Pseudomonas* medium). CFU values of bacterial isolates were assessed for mock-, *Chryseobacterium*-, and *Pseudomonas*-inoculated plants in each growth medium and expressed per unit of root fresh weight (CFU g^−1^) two days after incubation at 25  ±  1 °C. Four replicates (pool of five plants) with two technical replicates (plates) were analyzed for each inoculation condition (mock-, *Chryseobacterium*-, and *Pseudomonas*-inoculated plants) and temperature regime (non-stressed and cold-stressed plants).

### 4.5. Assessment of H_2_O_2_ Content in Tomato Shoots

H_2_O_2_ content was assessed in tomato shoots using the Amplex Red Hydrogen Peroxide/Peroxidase Assay Kit (Invitrogen, Thermo Fisher Scientific, Waltham, MA, USA) as previously described [46]. Briefly, each ground shoot sample (50 mg) was mixed with 250 µL of phosphate buffer (50 mM, pH 7.4), incubated in ice for 30 min under orbital shaking, and centrifuged at 12,000× *g* for 5 min at 4 °C. The supernatant (5 µL) was mixed with 45 µL of phosphate buffer (50 mM, pH 7.4) in a flat-bottom-black 96-well plate (Greiner 655076, Sigma-Aldrich, Merck). A calibration curve of H_2_O_2_ was obtained (0, 0.078, 0.156, 0.313, 0.625, 1.25, 2.5, and 5 μM) by mixing the H_2_O_2_ standard (10 µM) with phosphate buffer (50 mM, pH 7.4) in a final volume of 50 μL in each well. The reaction buffer (50 μL) containing the AmplexRed reagent and horseradish peroxidase (HRP) was added to each well with a total volume of 100 μL and mixed by pipetting. Samples were incubated in the dark at room temperature for 30 min, and fluorescence was measured using a Synergy 2 Multi-Mode Microplate Reader (Biotek, Winooski, VT, USA) with excitation range from 525 nm to 540 nm and emission range from 520 nm to 595 nm. Three replicates (pool of seven plants) and two technical replicates were analyzed for each inoculation condition and temperature regime.

### 4.6. Statistical Analysis

Fresh weight, dry weight, Log_10_-transformed CFU counts, and H_2_O_2_ content were analyzed with R version 4.3.0 (https://www.r-project.org/1, accessed on 1 February 2025). Normal distribution (Shapiro test, *p* > 0.05) and variance homogeneity (Levene test, *p* > 0.05) of the data were checked. When both assumptions were respected, the parametric *t*-test was used to detect significant differences (*p* ≤ 0.05) between bacterium-inoculated and mock-inoculated plants for each temperature regime and time point, or between cold-stressed and non-stressed samples for each inoculation condition. When parametric assumptions were not respected, the non-parametric Mann–Whitney test was used to detect significant differences (*p* ≤ 0.05) between bacterium-inoculated and mock-inoculated plants for each temperature regime and time point, or between cold-stressed and non-stressed samples for each inoculation condition.

### 4.7. RNA Extraction and Sequencing

Total RNA was extracted from 80 mg of ground sample using the Spectrum Plant Total RNA Kit (Sigma-Aldrich, Merck) with an on-column DNase treatment with RNase-Free DNase Set (Qiagen, Hilden, Germany). Total RNA quantity and quality were checked with a Qubit (Thermo Fisher Scientific) and a Tapestation 4150 (Agilent Technologies, Santa Clara, CA, USA), respectively. The success of DNase treatment was confirmed by PCR analysis with primers of a tomato housekeeping gene encoding ankyrin repeat domain containing protein 2 (*ARD2*; Table S3) in the absence of reverse transcription, and no amplification signals were detected. Three replicates (pool seven plants) were analyzed for each inoculation condition (mock-, *Chryseobacterium*-, and *Pseudomonas*-inoculated plants), temperature regime (non-stressed and cold-stressed plants), and time point (1 DAS and 14 DAS).

RNA samples were subjected to RNA-Seq library construction by external service at Fasteris (Plan-les-Ouates, Switzerland) using the TruSeq Stranded Total RNA with Ribo-Zero Plant library preparation kit (Illumina, SanDiego, CA, USA) according to the manufacturer’s instructions for an insert size of 120–200 bp. Quality controls were carried out according to the TruSeq Stranded Total RNA protocol (Illumina), samples were multiplexed, and paired-end reads of 150 nucleotides were obtained using a Novaseq instrument (Illumina) at Fasteris with a minimum sequencing depth of 5,870,000 paired-end reads per sample. Sequences were deposited at the Sequence Read Archive of the NCBI (https://www.ncbi.nlm.nih.gov/sra, accessed on 27 August 2025) under the BioProject number PRJNA1079540.

### 4.8. Bioinformatic Analysis, Identification, and Functional Annotation of Differentially Expressed Genes

After assessing the quality of the RNA-Seq raw reads with FastQC v0.11.5 (https://www.bioinformatics.babraham.ac.uk/projects/fastqc/, accessed on 27 August 2025), adapter sequences and low-quality reads were removed using Fastq-mcf v1.05 with the settings as follows: minimum quality threshold of 30 and a minimum read length of 50 base pairs. Filtered read pairs were mapped to the *S. lycopersicum* reference genome version ITAG5.0 (https://phytozome-next.jgi.doe.gov/, accessed on 27 August 2025) [47] using STAR version 2.7.11 with default parameters, and read counts of each tomato gene were assessed using htseq-count version 1.99.2 with default parameters. Read counts were normalized according to the library dimension, and gene expression levels were calculated as reads per million mapping to the gene (RPM). A gene was considered active if RPM values were greater than one in at least one sample, and non-active genes were filtered out. PCA was obtained in R using the *PCAtools* function, version 2.20.0.

Differential expressed genes (DEGs) were identified with the DESeq2 package version 1.47.1 in R with default parameters [48] imposing a FDR lower than 0.05 (adjusted *p*-value ≤ 0.05) and a LFC higher than 1 or lower than −1 in the pairwise comparisons between the cold-stress and non-stress conditions of mock-inoculated plants (cold-stressed mock-inoculated vs. non-stressed mock-inoculated), *Chryseobacterium*-inoculated plants (cold-stressed Chryseobacterium-inoculated vs. non-stressed *Chryseobacterium*-inoculated), and *Pseudomonas*-inoculated plants (cold-stressed Pseudomonas-inoculated vs. non-stressed *Pseudomonas*-inoculated) for each time point (1 DAS and 14 DAS). The distribution of DEGs was summarized using the Venn diagram (http://bioinformatics.psb.ugent.be/webtools/Venn/, accessed on 27 August 2025). DEGs were grouped into genes upregulated or downregulated by cold stress in all inoculation conditions (groups G4, G8, G12, and G16) and those upregulated or downregulated exclusively by cold stress in mock- (groups G3, G7, G11, and G15), *Chryseobacterium*- (groups G1, G5, G9, and G13), or *Pseudomonas*- (groups G2, G6, G10, and G14) inoculated plants.

### 4.9. Functional Annotation of Differentially Expressed Genes

Protein descriptions and Gene Ontology (GO) annotations of each DEG were obtained from the tomato reference genome (*S. lycopersicum* version ITAG5.0; https://phytozome-next.jgi.doe.gov/, accessed on 27 August 2025) [47]. GO enrichment analysis was performed in R using the *topGO* package version 2.58.0 on genes upregulated or downregulated by cold stress in all inoculation conditions (groups G4, G8, G12, and G1, or exclusively modulated in mock- (groups G3, G7, G11, and G15), *Chryseobacterium*- (groups G1, G5, G9, and G13), or *Pseudomonas*- (groups G2, G6, G10, and G14) inoculated plants, and enriched categories belonging to biological processes were identified using the *classic* algorithm with Fisher’s test (*p* ≤ 0.05) [49].

DEGs were further annotated according to protein descriptions obtained from a homology search against SwissProt and TREMBL protein databases (downloaded in February 2025; https://www.uniprot.org/, accessed on 27 August 2025) using Diamond v2.1.9 with the BLASTX mode. The results were integrated, and the GO terms were assigned to the protein sequences using the AHRD tool v3.11. Heatmaps summarizing the expression profiles and putative functions of DEGs belonging to functional categories of oxidative stress, stress response, and hormonal signaling were generated.

### 4.10. Gene Expression Analysis by Quantitative Real-Time PCR

The first strand cDNA was synthesized from total RNA (1 μg) with the Superscript III (Invitrogen, Thermo Fisher Scientific) and oligo-dT primer. *NCED5*, *CBF1*, *CHS2*, *HY5*, *F3’5’H*, *GST*, and *SUS* genes were used for RNA-Seq validation, since they are known gene markers modulated during tomato response to cold stress [14,28,39] (Table S3). qPCR reactions were carried out with specific primers (Table S3) using the Light Cycler 480 (Roche, Merck, Rahway, NJ, USA) and the Platinum SYBR Green qPCR SuperMix-UDG (Invitrogen, Thermo Fisher Scientific), as previously described [50]. Each sample was examined in three technical replicates, and dissociation curves were analyzed to verify the specificity of each amplification reaction (PCR conditions: 50 °C for 2 min and 95 °C for 2 min as initial steps, followed by 50 cycles at 95 °C for 15 s and at 60 °C for 1 min). The Ct values were calculated with the Light Cycler 480 SV 1.5.0 software (Roche) according to the second derivative, and the reaction efficiency (Eff) was assessed for each gene with the LinRegPCR 11.1 software [51]. The expression levels were calculated according to the Hellemans equation [52], using housekeeping genes encoding *ARD2* [53] and glyceraldehyde-3-phosphate dehydrogenase (*GADPH*) [27] (Table S3). For each temperature regime and time point, relative quantities (RQ) were calculated according to the following formula: RQ = Eff^(Ct–Ct′)^, where Ct is the threshold cycle and Ct’ is the mean Ct value of mock-inoculated plants. Normalized relative quantities (NRQ) were then obtained by dividing the RQ values by the normalization factors (i.e., RQ values of the two housekeeping genes) [52]. Three replicates (pool of seven plants) were analyzed for each inoculation condition, temperature regime, and time point.

## 5. Conclusions

*Chryseobacterium* sp. GRCS301 and *Pseudomonas* sp. GRCS202 isolated from alpine plants promoted shoot growth and decreased oxidative stress in tomato plants at low temperature. Transcriptomic analysis revealed that plants inoculated with these cold-tolerant bacterial endophytes upregulated key stress-related pathways under cold-stress conditions, such as genes involved in ROS homeostasis, cold-stress response, and hormonal signaling. These results highlight the potential of selected bacterial inoculants to enhance cold-stress acclimation in tomato plants as a sustainable tool to improve crop resilience to low temperatures.

## Data Availability

Raw RNA reads were deposited at the Sequence Read Archive of the NCBI (https://www.ncbi.nlm.nih.gov/sra, accessed on 27 August 2025) under the BioProject number PRJNA1079540.

## Supplementary Materials

The following supporting information can be downloaded at https://www.mdpi.com/article/10.3390/plants14213316/s1, Figure S1: Diagram of plant growth conditions and sampling time points; Figure S2: Bacterial re-isolation from tomato rhizosphere under non-stress and cold-stress conditions; Figure S3: Principal component analysis (PCA) of RNA-Seq data. Figure S4: RNA-Seq data validation by qPCR; Table S1. Taxonomic annotation of cold-tolerant bacterial isolates; Table S2. Expression levels of tomato genes; Table S3. Primer sequences of tomato genes analyzed by quantitative real-time PCR; Table S4. Expression levels and functional annotations of differentially expressed genes.
