# Supplementary material for: Cold-Tolerant Bacteria Isolated from Alpine Plants Can Promote Growth and Mitigate Cold Stress in Tomato Seedlings by Complex Transcriptional Reprogramming of Stress-Related Genes
Source: Plants (Basel). 2025 Oct 30;14(21):3316. doi: 10.3390/plants14213316 (PMC12610570; doi:10.3390/plants14213316)
Supplement: Supplementary file 1 [file plants-14-03316-s001.zip › Supplementary Figures.pdf]

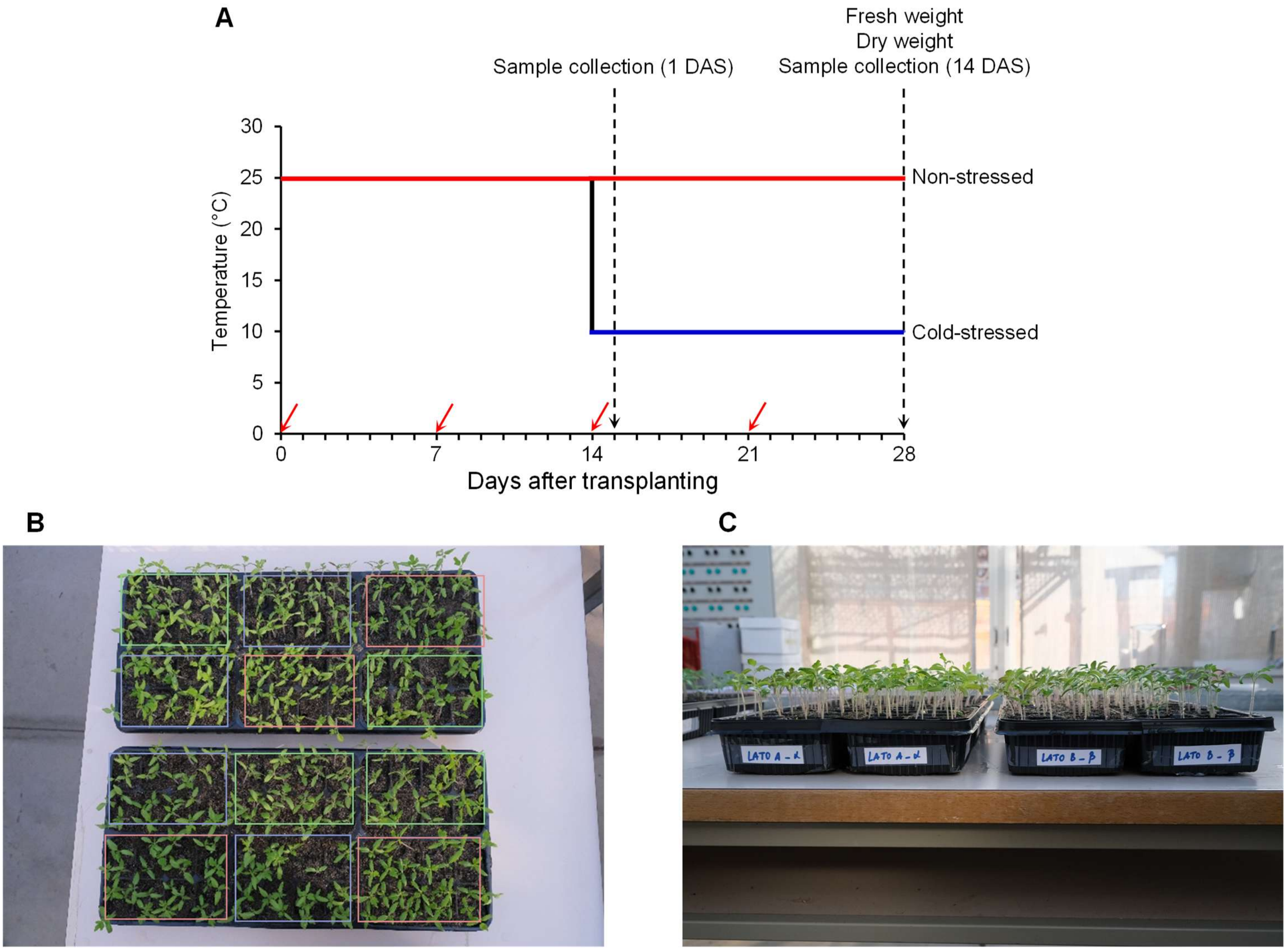

Figure S1. Diagram of plant growth conditions and phenotype of plants. Germinated seeds were transplanted in pots containing a sterilized soil:sand mixture and grown in a growth chamber at 25 ± 2 °C with a 14:10 light:dark photoperiod (A). Plants were treated at the root collar with $MgSO_4$ (mock-inoculated) or inoculated with the respective cold-tolerant bacterial isolate (bacterium-inoculated) every seven days starting from the transfer into the soil:sand mixture (0, 7, 14, and 21 days after transplanting; red arrows) for a total of four applications using a randomized block design. In the screening trials, mock-inoculated and bacterium-inoculated plants were grown for 28 days at 25 ± 2 °C with a 14:10 light:dark photoperiod. In the validation trials and functional characterization trials, mock-inoculated and bacterium-inoculated plants were grown for 14 days in the growth chamber at 25 ± 2 °C with a 14:10 light:dark photoperiod, and two groups of plants were then obtained, and they were incubated at 25 ± 2 °C in the growth chamber (non-stressed plants) or at 10 ± 2 °C in another growth chamber (cold-stressed plants) for 14 days with a 14:10 light:dark photoperiod. Four replicates (screening trials and validation trials) or three replicates (functional characterization trials) were analyzed for each inoculation condition and temperature regime, and each replicate consisted of a pool of four plants (screening trials) or seven plants (validation trials and functional characterization trials). Shoot fresh weight (screening trials, validation trials, and functional characterization trials) and dry weight (screening trials and validation trials) were assessed

at the end of the incubation in the growth chamber. In the functional characterization trials, shoots of mock-, *Chryseobacterium*-, and *Pseudomonas*-inoculated plants were collected at one day and 14 days after stress exposure (DAS; dotted arrows) from non-stressed and cold-stressed plants for H_2O_2 analysis (1 DAS), RNA-seq analysis (1 DAS and 14 DAS), and bacterium re-isolation (14 DAS). Examples of the plant phenotype of plants treated with $MgSO_4$ (mock-inoculated; green) or inoculated with *Chryseobacterium* sp. GRCS301 (blue) or *Pseudomonas* sp. GRCS202 (red), and incubated under non-stress (B, upper tray; and C, left tray) and cold-stress (B, lower tray; and C, right tray) conditions are reported.

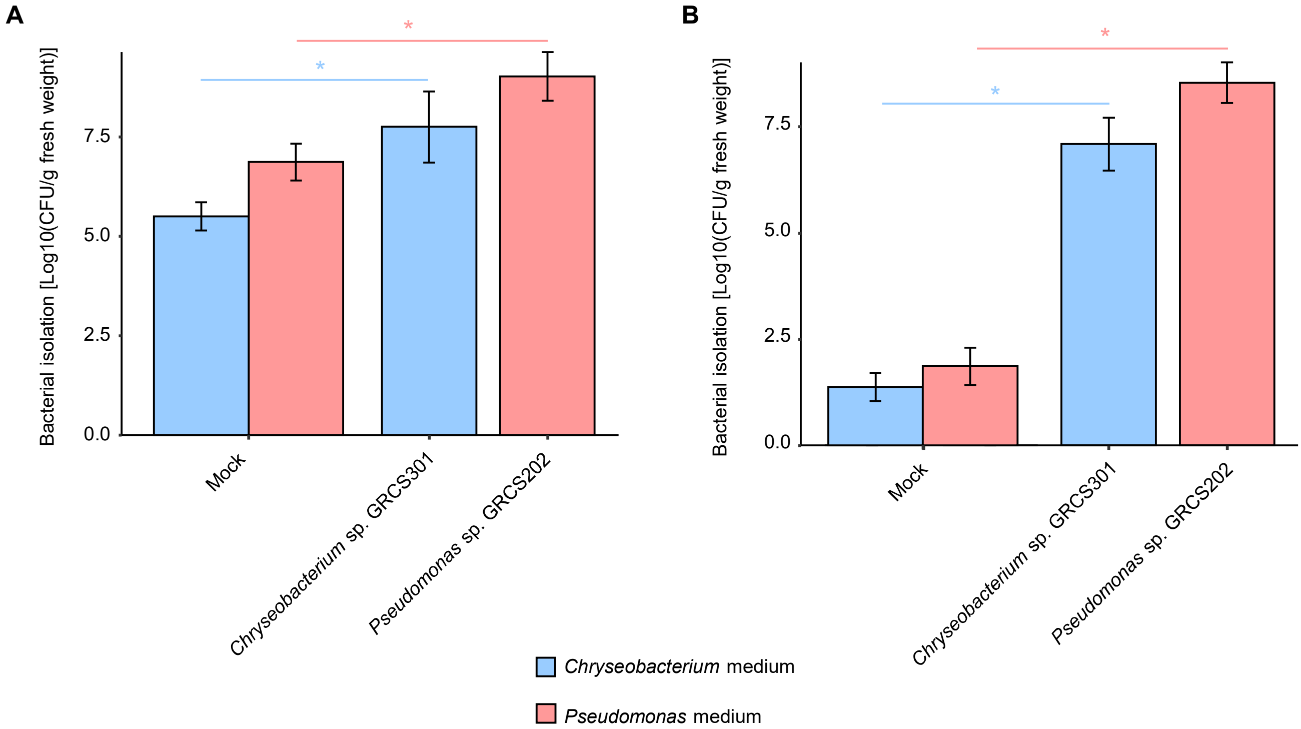

Figure S2. Bacterial re-isolation from tomato rhizosphere under non-stress and cold-stress conditions. Tomato plants were treated with $MgSO_4$ (mock-inoculated) or inoculated with *Chryseobacterium* sp. GRCS301 or *Pseudomonas* sp. GRCS202, and rhizosphere samples were collected at the end of incubation at 25 ± 2 °C (non-stressed plants; A) or at 10 ± 2 °C (cold-stressed plants; B). Colony forming units (CFU) of rhizosphere bacteria were assessed on semi-selective R2A media to allow *Chryseobacterium* spp. (*Chryseobacterium* medium) and *Pseudomonas* spp. (*Pseudomonas* medium) growth. Mean Log_{10}-transformed CFU and standard error values of four replicates (pool of five plants) with two technical replicates are reported for each inoculation condition, temperature regime, and growth medium. Asterisks indicate significant differences between bacterium-inoculated and mock-inoculated samples for each temperature regime and growth medium, according to the t-test ($P \leq 0.05$).

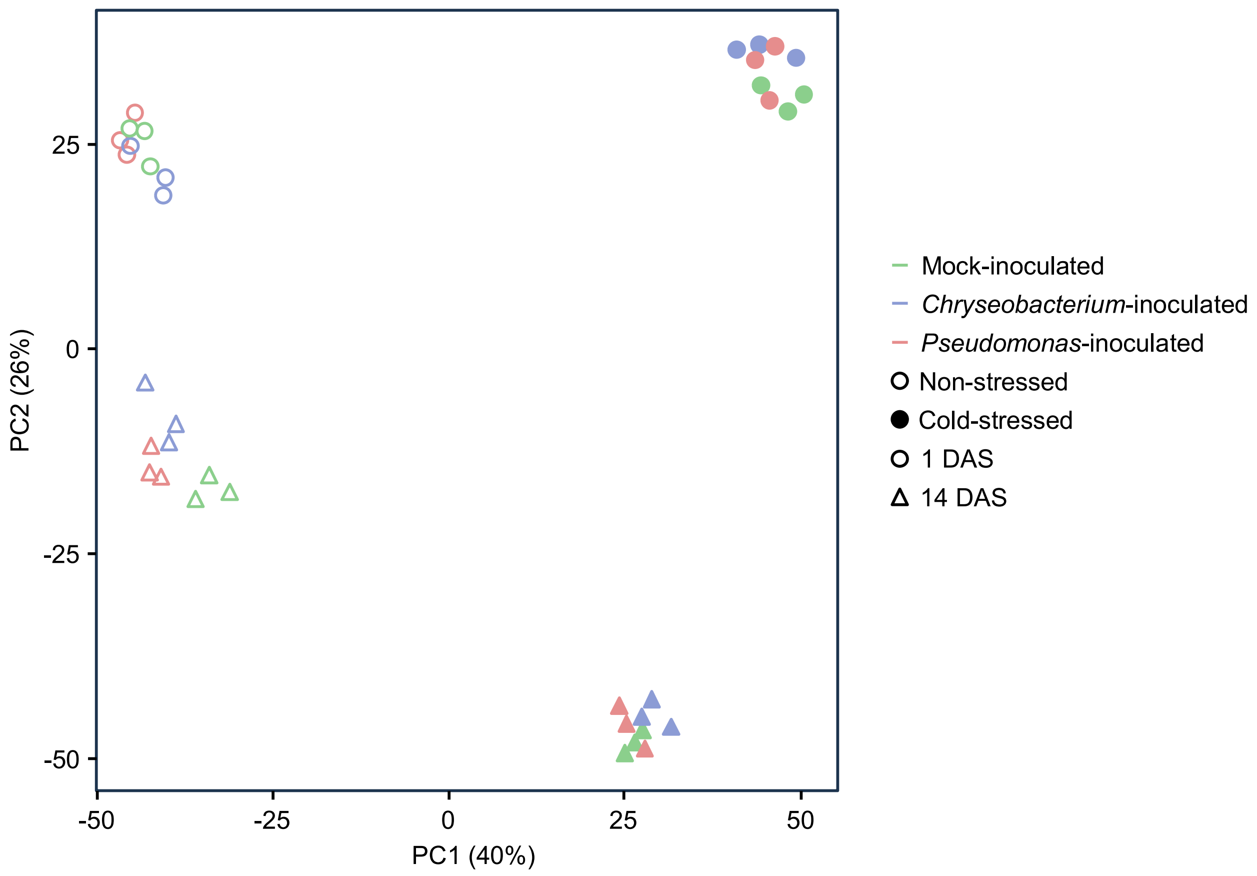

Figure S3. Principal component analysis (PCA) of RNA-Seq data. Tomato plants were treated with $MgSO_4$ (mock-inoculated) or inoculated with *Chryseobacterium* sp. GRCS301 (*Chryseobacterium*-inoculated) or *Pseudomonas* sp. GRCS202 (*Pseudomonas*-inoculated), and shoot samples were collected at one day (1 DAS) and 14 days (14 DAS) after stress exposure (DAS) from plants incubated at 25 ± 2 °C (non-stress condition) or at 10 ± 2 °C (cold-stress condition). PCA plot was obtained on normalized counts of 21,698 active genes.

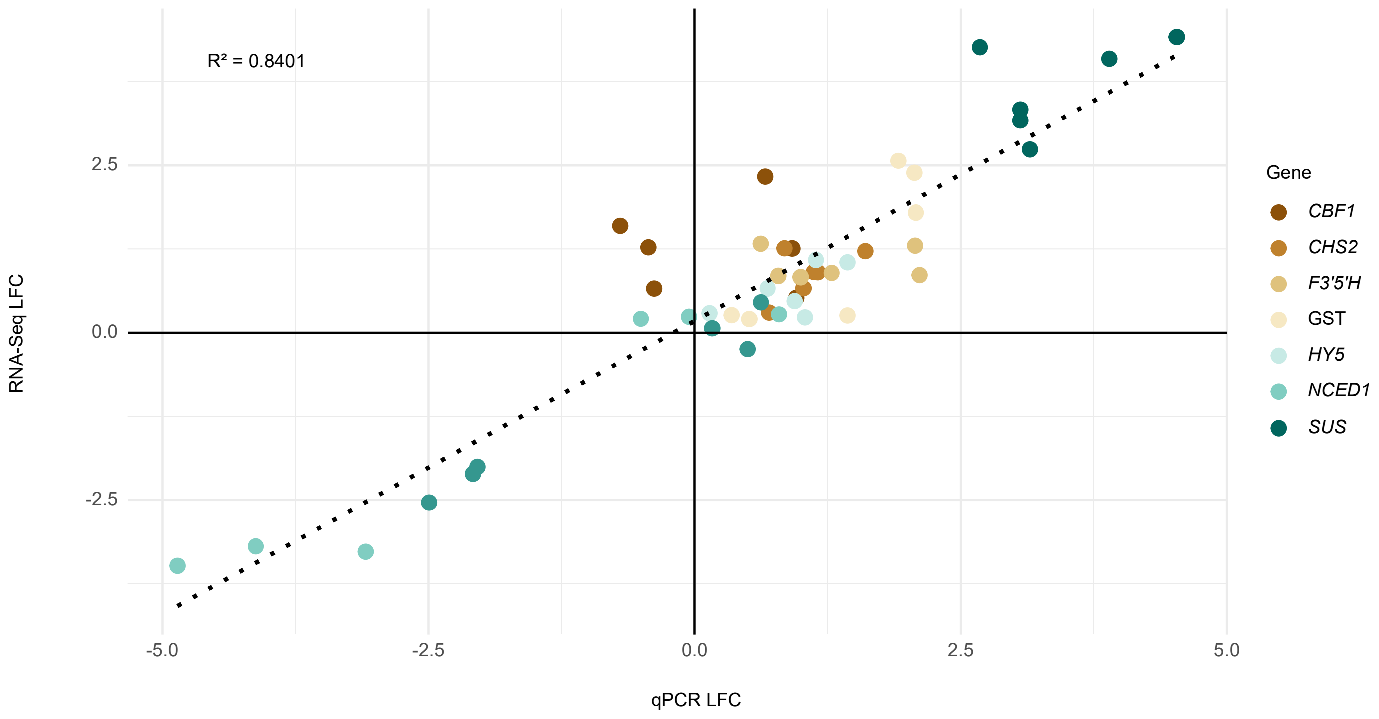

Figure S4. RNA-Seq data validation by qPCR. Scatter plot of Log_2-transformed fold change (LFC) values assessed by RNA-Seq and qPCR analyses is reported together with the regression line equation and the R^2 coefficient based on expression levels of genes encoding the 9-cis-epoxycarotenoid dioxygenase (*NCED5*), C-repeat/DRE binding factor 1 (*CBF1*), chalcone synthase (*CHS2*), elongated hypocotyl 5 transcriptional factor (*HY5*), flavonoid-3'-hydroxylase (*F3'5'H*), glutathione-S-transferase enzyme (*GST*), and sucrose synthase (*SUS*) in shoot samples of tomato plants treated with $MgSO_4$ (mock-inoculated), inoculated with *Chryseobacterium* sp. GRCS301 or *Pseudomonas* sp. GRCS202, and incubated at 25 ± 2 °C (non-stress condition) or at 10 ± 2 °C (cold-stress condition) for 14 days, and collected at one day and 14 days after stress exposure (DAS). Gene annotations are indicated by the colored legend.

Table S1. Taxonomic annotation of cold-tolerant bacterial isolates.

Taxonomic annotations (columns A-F) are reported for each cold-tolerant bacterium previously isolated from the roots of three alpine Rosaceae plants (*Alchemilla sp., Dryas octopetala*, and *Geum montanu*; Marian et al. 2025, doi: 10.1128/mbio.01418-24) and specified by the isolate code (column G) and accession number (column H) of the National Center for Biotechnology Information (NCBI; http://www.ncbi.nlm.nih.gov). Accession numbers (column I), identity percentages (column J), and taxonomic annotations (column K) are reported for the best ten hits of the nucleotide alignment against the 16S ribosomal RNA database of the NCBI.

Table S2. Expression levels of tomato genes. For each tomato gene (column A), protein description (column B), Gene Ontology category (column C) from the reference genome (*S. lycopersicum* version ITAG5.0; https://phytozome-next.jgi.doe.gov/), and protein description obtained by homology search against SwissProt and TREMBL protein databases (column D) are reported. Expression levels are reported as reads per million (RPM) for each shoot sample of tomato plants treated with MgSO4 (mock-inoculated) or inoculated with *Chryseobacterium* sp. GRCS301 (*Chryseobacterium*-inoculated) or *Pseudomonas* sp. GRCS202 (*Pseudomonas*-inoculated), and collected at one day (1 DAS) and 14 days (14 DAS) after stress exposure (DAS) from plants incubated at 25 ± 2 °C (non-stress condition; NS) or at 10 ± 2 °C (cold-stress condition; CS) in triplicate (named R1, R2, and R3). Mean values (columns E-P), standard deviation values (columns Q-AB), and values of each replicate (columns AC-BK) are reported.

Table S3. Primer sequences of tomato genes analyzed by quantitative real-time PCR. Gene name (column A), gene abbreviation (column B), and tomato gene identifier (column C; *S. lycopersicum* reference genome ITAG5.0 https://phytozome-next.jgi.doe.gov/) are reported for the selected tomato genes. Sequences of the forward primer (column D) and reverse primer (column E) are indicated for each gene with the respective bibliographic reference (column F).

Table S4. Expression levels and functional annotations of differentially expressed genes. Tomato plants were treated with MgSO4 (mock-inoculated) or inoculated with *Chryseobacterium* sp. GRCS301 (*Chryseobacterium*-inoculated) or *Pseudomonas* sp. GRCS202 (*Pseudomonas*-inoculated), and shoot samples were collected at one day (1 DAS) and 14 days (14 DAS) after stress exposure (DAS) from plants incubated at 25 ± 2 °C (non-stress condition; NS) or at 10 ± 2 °C (cold-stress condition; CS). Differentially expressed genes (DEGs) were selected imposing a false discovery rate (FDR) lower than 0.05 (adjusted *P*-value ≤ 0.05) and a Log_2-transformed fold change (LFC) higher than 1 or lower than -1 in the pairwise comparisons between the cold-stress and non-stress conditions for each inoculation condition (cold-stressed mock-inoculated *vs* non-stressed mock-inoculated, cold-stressed *Crhyseobacterium*-inoculated *vs* non-stressed *Crhyseobacterium*-inoculated, and cold-stressed *Pseudomonas*-inoculated *vs* non-stressed *Pseudomonas*-inoculated) and each time point (1 DAS and 14 DAS). For each DEG (column A), LFC values (columns B-G) and *P*-values (columns H-M) are reported for each pairwise comparison. For each time point, DEGs were grouped (column N) in genes upregulated or downregulated in CS samples compared to NS samples in all inoculation conditions (groups 4, 8, 12, 16) or exclusively in mock-inoculated inoculated (groups 3, 7, 11, 15), *Chryseobacterium*-inoculated plants (groups 1), or *Pseudomonas*-inoculated

plants (groups 2, 6, 10, 14), according to the Venn diagrams (Figure 2). Protein description (column O), Gene Ontology category (column P) from the reference genome (*S. lycopersicum* version ITAG5.0; https://phytozome-next.jgi.doe.gov/), and protein description obtained by a homology search against SwissProt and TREMBL protein databases (column Q) are reported for each gene. According to the protein descriptions, DETs were assigned to functional categories of oxidative stress, stress response, and hormonal signaling (columns R-S).
